# Reference Values for B Vitamins in Human Milk: The Mothers, Infants and Lactation Quality (MILQ) Study

**DOI:** 10.1016/j.advnut.2025.100500

**Published:** 2025-10-28

**Authors:** Lindsay H Allen, Setareh Shahab-Ferdows, Sophie E Moore, Janet M Peerson, Gilberto Kac, Amanda C Figueiredo, Daphna K Dror, Kim F Michaelsen, M Munirul Islam, Fanta Nije, Daniela Hampel, MILQ Study Consortium, Lindsay H Allen, Sophie E Moore, Gilberto Kac, Kim F Michaelsen, Christian Mølgaard, M Munirul Islam, Maria Andersson, Setareh Shahab-Ferdows, Sophie H Christensen, Jack I Lewis, Janet M Peerson, Xiuping Tan, Daphna K Dror, Andrew M Doel, Daniela de Barros Mucci, Bruna C Schneider, Farhana Khanam, Adriana Divina de Souza Campos, Gabriela Torres Silva, Fanta Nije, Mehedi Hassan, Amanda C Figueiredo, Daniela Hampel

**Affiliations:** 1 USDA, ARS Western Human Nutrition Research Center, University of California Davis, Davis, CA, United States; 2 Institute for Global Nutrition, Department of Nutrition, University of California, Davis, CA, United States; 3 Medical Research Council Unit The Gambia at London School of Hygiene & Tropical Medicine, Fajara, The Gambia, West Africa; 4 Department of Women and Children’s Health, King's College London, London, United Kingdom; 5 Nutritional Epidemiology Observatory, Josué de Castro Nutrition Institute, Federal University of Rio de Janeiro, Rio de Janeiro, RJ, Brazil; 6 Department of Nutrition, Exercise and Sports, Faculty of Science, University of Copenhagen, Copenhagen, Denmark; 7 Nutrition and Clinical Services Division, International Centre for Diarrhoeal Disease Research, Bangladesh (icddr,b), Dhaka, Bangladesh

**Keywords:** human milk, lactation, B vitamins, reference values, human milk nutrient concentration, infant nutrition

## Abstract

This third article in the series presenting reference values (RVs) for nutrients in human milk describes the values for B vitamins. The mothers, infants, and lactation quality (MILQ) and early-MILQ studies, conducted at sites in Bangladesh, Brazil, Denmark, and The Gambia, were designed to measure human milk nutrient concentrations of well-nourished mothers during the first 8.5 mo of lactation. Applying ultrahigh-performance liquid chromatography–mass spectrometry (UPLC-MS/MS) to analyze multiple B vitamins simultaneously produced RVs for vitamin B2, B3, pantothenic acid, B6, and biotin. Choline was analyzed separately by UPLC-MS/MS, vitamin B1 by high-performance liquid chromatography–fluorescence detection, and vitamin B12 by competitive chemiluminescence enzyme immunoassay. Measured milk B-vitamin concentrations from the MILQ study were compared with those used by the Institute of Medicine (IOM) for setting recommendations for nutrient requirements of infants. MILQ estimates were substantially lower (<60% of the concentrations used by the IOM) for vitamins B1, B2, and B6; 60%–100% of concentrations used by the IOM for vitamin B3, vitamin B12, and choline; and consistent or slightly (100%–125%) higher than concentrations used by the IOM for pantothenic acid and biotin. Total daily median B-vitamin intakes from 1 to 6 mo were 29%–45% of IOM adequate intakes (AIs) for vitamins B1 and B2, 60%–75% of AIs for vitamins B3, B6, B12, and choline, and 118%–128% of AIs for pantothenic acid and biotin. The MILQ B-vitamin concentrations are provided as percentile curves to enable comparison and interpretation of data from other studies.

**Statement of significance**

The mothers, infants and lactation quality study overcame the major limitations of previous studies on B vitamins in human milk by validating the methods of analysis and excluding maternal vitamin supplementation. Most concentrations and, therefore, levels of intake of B vitamins were lower than those assumed by the Institute of Medicine when establishing recommended intakes for infants, indicating that current recommendations for infants’ requirements of most B vitamins may be higher than required. The reference values can be used to determine the need for and efficacy of intervention such as supplementation.

## Introduction

The mothers, infants and milk quality (MILQ) study was designed to develop reference values (RVs) for nutrient concentrations in the milk of well-nourished mothers. The study design and methods have been described in detail previously [1,2]. In brief, human milk samples and anthropometric, dietary, biochemical, and other data were collected from mother–infant dyads at 4 study sites (Bangladesh, Brazil, Denmark, and The Gambia) where foods are not industrially fortified with B vitamins. Collection occurred from 0 to 1 mo [early milk, or early-MILQ (E-MILQ), with sample collection at 4–17 d (E1) and 18–31 d (E2)], then 3 times between 1 and 8.5 mo lactation [1–3.49 mo (M1), 3.5–5.99 mo (M2), and 6–8.5 mo (M3)].

The application of liquid chromatography-mass spectrometry (LC-MS) for measuring human milk composition has been particularly valuable for quantifying B vitamins [3]. There are few studies with data on the concentrations of B vitamins in milk due to difficulties or uncertainties about measuring the different vitamers [4,5]. Additional challenges include the fact that there are 8 B vitamins (including choline), and the influence of recent maternal diet and/or status and time postpartum on their secretion into milk [4,6]. In this article, the vitamins are referred to by number rather than name, because the named vitamin is often only 1 of the forms.

Folate concentrations were not measured because folate requires a different analytical procedure from the other B vitamins, most of which could be measured together. Maternal factors, including status and diet, are known not to affect folate concentrations in milk among healthy, well-nourished women [7].

Lack of reliable data on nutrient concentrations in human milk and volumes of infant intake limits the Institute of Medicine’s (IOM’s) ability to set accurate nutrient recommendations for infants, including estimated average requirement (EAR), and recommended dietary allowances (RDAs) or adequate intakes (AIs). Of note, the IOM was renamed the National Academy of Medicine (NAM) in 2015, but since dietary reference intakes for B vitamins were published prior to the change we will refer to the institute as the IOM. Currently, for infants, IOM micronutrient recommendations are all AI values, reflecting the insufficient data required to establish the more robust RDA values. Thus, the infants’ recommended intakes of B-vitamins (and other nutrients) have typically been calculated as the mean intakes of full-term infants exclusively breastfed by well-nourished mothers during the first 6 mo [8]. This calculation is based on the mean concentration of the vitamins from 2 to 6 mo of lactation using estimated values from several reported studies and an assumed mean milk intake of 0.78 L/d [8]. Depending on the vitamin in question, the studies used for setting the AIs included as few as 5 women (riboflavin) and ≤111 women (vitamin B12) and were conducted between 1951 and 1997 [8]. Thus, the current AIs are based on data from outdated studies with few participants, indicating the need for revision. Additional problems with existing data include inconsistent timing and methods of milk collection, invalid analytical methods, different stages of lactation, and a lack of information about maternal diet and any maternal supplements [9].

Methods used for analyzing B-vitamins have historically been derived from those developed for other matrices, such as plasma or urine, and were not necessarily validated for B-vitamin analysis in the complex human milk matrix [10]. Results obtained with different methods have been inconsistent, probably due to the effect of milk constituents, including sugars and fat.

MILQ and E-MILQ results are compared with currently accepted values (mostly those accepted by the IOM for deriving intake recommendations) and those reported elsewhere, emphasizing the relatively few recent reviews. Older values should be accepted with caution due to possibly inaccurate analytical methods. The graphs show the concentration of each nutrient in milk by site and day of lactation, and percentiles by day of lactation. Resulting RVs for B vitamins are presented in this article as median concentrations for study sites combined (Table 1) and as percentile curves. In addition, total daily intakes of each B vitamin were calculated as concentration × milk volume for each mother–infant dyad at each time point (Table 2). Supplementary tables include estimated percentile values of nutrient concentration by month postpartum (Supplemental Table 1) as well as median total nutrient intake by study visit (1–3.49 mo, 3.5–5.99 mo, 6–8.5 mo) (Supplemental Table 2). The introductory article in this series [2] describes how the graphs and values were constructed, the flow diagrams, and the main reasons for sample exclusion during MILQ study visits. These included infant age-for-weight, age-for-height, or weight-for-length *Z*-scores < −2 and cessation of exclusive breastfeeding by the first MILQ study visit (1–3.49 mo) [2].

**TABLE 1:** Comparison of MILQ (1–6 mo) and IOM values (0–6 mo) for B-vitamin concentrations in human milk.

Nutrient	MILQ^1^ median	MILQ IQR	IOM	MILQ as % IOM
B1 (μg/L)	111	(87–139)	210	53
B2 (μg/L)	115	(80–155)	350	33
B3 (mg/L)	1.4	(1.0–1.9)	1.8	78
Pantothenic acid (mg/L)	2.4	(1.8–3.1)	2.2	109
B6 (μg/L)	76	(48–112)	130	58
Biotin (μg/L)	8	(5–11)	6	125
B12 (μg/L)	0.29	(0.22–0.44)	0.42	69
Choline (mg/L)	109	(87–135)	160	68

Abbreviations: IOM, Institute of Medicine; MILQ, mothers, infants, and lactation quality study.

1 MILQ values are pooled median concentrations from 1 to 6 mo.

**TABLE 2:** Comparison of MILQ pooled median B-vitamin intake in exclusively breastfed infants from 1 to 6 mo with IOM adequate intakes.

Nutrient	MILQ	IOM AI	% AI
B1 (μg/d)	90	200	45
B2 (μg/d)	87	300	29
B3 (mg/d)	1.2	2.0	60
Pantothenic acid (mg/d)	2.0	1.7	118
B6 (μg/d)	65	100	65
Biotin (μg/d)	6.4	5.0	128
B12 (μg/d)	0.25	0.40	63
Choline (mg/d)	94	125	75

Abbreviations: AI, adequate intake; IOM, Institute of Medicine; MILQ, mothers, infants, and lactation quality study.

## Vitamin B1

### Background

Vitamin B1, in its active form thiamine pyrophosphate (TPP), is essential for brain development and for maintaining normal functioning of the cardiovascular and nervous systems. TPP is also a cofactor for enzymes involved in carbohydrate and fat metabolism [11]. Because physiological storage is limited, humans depend on regular dietary intake to meet their needs [8]. Higher milk than plasma concentrations of vitamin B1 suggest an active secretion process [12,13]. Maternal vitamin B1 intake is positively associated with milk concentration regardless of maternal status [9]. Although vitamin B1 is preferentially transported into milk in the case of maternal deficiency [9], poor maternal status and/or intake and subsequently low milk vitamin B1 is a leading cause of infantile beriberi [14]. Vitamin B1 depletion is more likely where diets are high in refined grains and thiaminases and low in animal-source foods and legumes, such as in Southeast Asia [15,16].

### Methods

Approximately 70% of vitamin B1 in human milk is present as thiamine monophosphate (TMP) and ∼30% as thiamine [13]. Vitamin B1 was analyzed by HPLC—fluorescence detection, which quantified the vitamer forms thiamine, TMP, and TPP [13,17].

### Results

**FIGURE 1:**
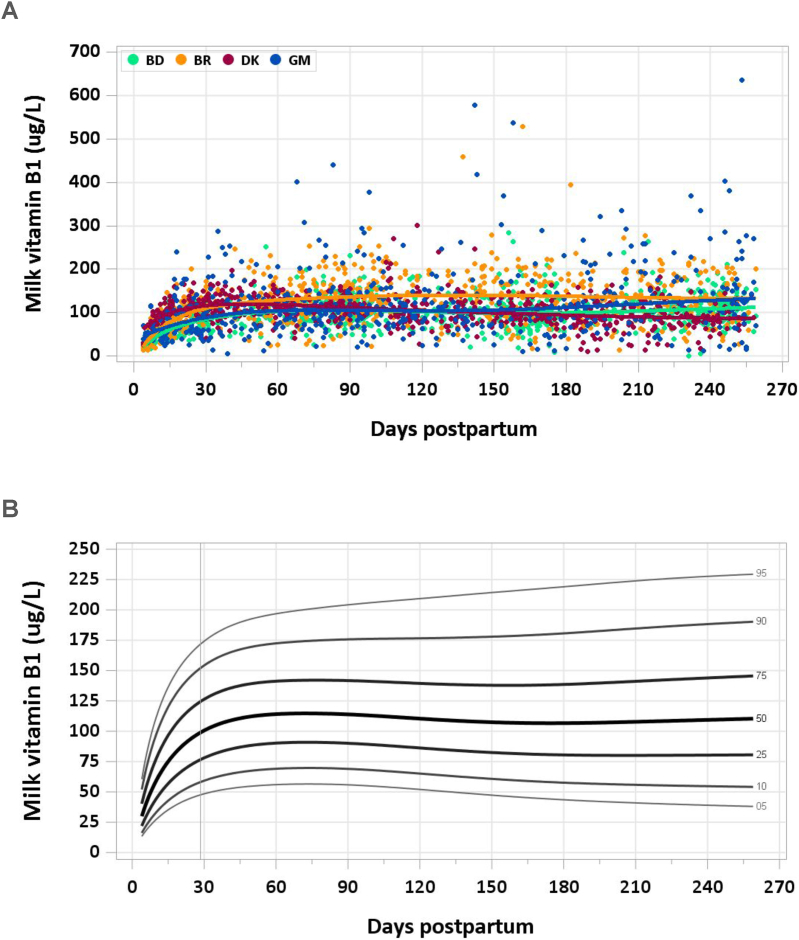
(A) Distribution of human milk vitamin B1 concentrations. (B) Percentile curves for vitamin B1 concentration in human milk.

A total of 3072 samples were analyzed (2498 MILQ, 574 E-MILQ), of which 2 were removed for implausible values (2 MILQ, 0 E-MILQ), and 305 for not meeting inclusion criteria (253 MILQ, 52 E-MILQ), for a total included sample size of 2765 (2243 MILQ, 522 E-MILQ). Median milk vitamin B1 concentration increased from 63.9 μg/L at 4–17 d to 96.9 μg/L at 18–31 d. Median milk vitamin B1 peaked at 114.5 μg/L from 2 to 3 mo, then decreased slightly to a steady concentration of 107–110 μg/L from 4 mo through the end of the study period (Figure 1A, B). The pooled median total vitamin B1 intake from 1 to 6 mo was 90 μg/d.

### Comparison with published values

Referring to the Committee on Nutrition’s 1985 Pediatric Nutrition Handbook [18], the IOM in 1998 assumed a mean concentration of vitamin B1 in mature human milk of 210 μg/L. Multiplied by a mean milk intake of 0.78 L/d, the AI was calculated as 160 μg/d but rounded to 200 μg/d (0.2 mg) [8]. The median concentration of vitamin B1 in mature milk in the present study is ∼55% of the concentration used by the IOM when determining nutrient recommendations. It is ∼60% of a suggested concentration of 190 μg/L calculated as 90% of maximal milk vitamin B1 concentrations achieved by supplementing 335 healthy women in Cambodia from 2 to 24 wk postpartum [19]. In another short-term supplementation trial, baseline median Cambodian milk vitamin B1 concentration was significantly lower (60 μg/L) than that of thiamine-replete American mothers (169 μg/L) at 4 mo postpartum. Five daily doses of 100 mg thiamine hydrochloride increased the median milk vitamin B1 concentration of Cambodian mothers to 170 μg/L [20]. We conclude that the supplementation trials increased milk vitamin B1 to levels substantially above what can be used as an RV.

## Vitamin B2

### Background

Vitamin B2 (commonly called riboflavin) is the precursor of the coenzymes flavin mononucleotide (FMN) and flavin adenine dinucleotide (FAD), both of which act as electron carriers involved in oxidation–reduction reactions that are necessary for energy production and antioxidant function [9]. A global analysis of micronutrient intakes showed that 55% of the world population has a dietary inadequacy of vitamin B2, with the highest prevalence of inadequate intakes in South Asia and Africa [21]. Vitamin B2 deficiency affects many metabolic pathways and can result in peripheral neuropathy, poor growth, and impaired iron absorption [22]. The concentration of the vitamin in human milk correlates with maternal status in most studies, and supplementation increases milk concentrations [9]. Vitamin B2 concentrations in milk respond rapidly to maternal intake [[23], [24], [25]], and status is often a reflection of the amounts of dairy products consumed [26,27].

### Methods

Most vitamin B2 in milk is FAD and riboflavin, with some FMN [28]. In the present study, vitamin B2 in milk was measured by ultrahigh-performance liquid chromatography-mass spectrometry (UPLC-MS/MS), which quantified the vitamers riboflavin, FAD, and FMN [17].

### Results

**FIGURE 2:**
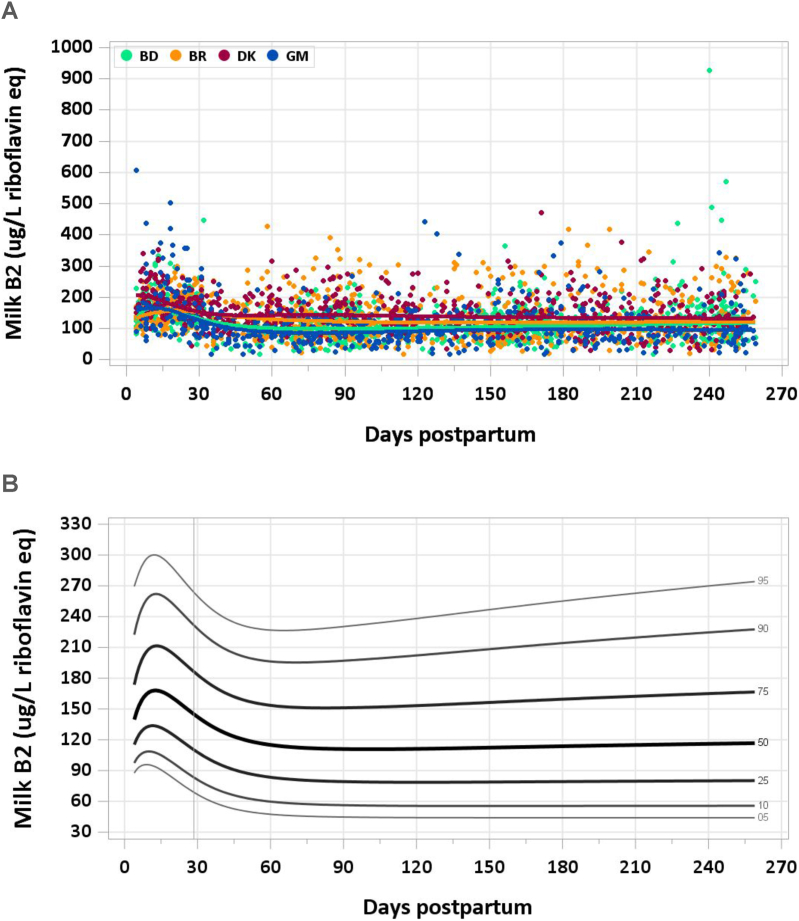
(A) Distribution of human milk vitamin B2 concentrations. (B) Percentile curves for vitamin B2 concentration in human milk.

For vitamins B2, B3, and pantothenic acid, a total of 3080 samples were analyzed (2506 MILQ, 574 E-MILQ), of which none were removed for implausible values and 306 for not meeting inclusion criteria (254 MILQ, 52 E-MILQ), for a total included sample size of 2774 (2252 MILQ, 522 E-MILQ). Median concentrations were 165 μg/L at both 4–17 d and 18–31 d, although these pooled medians likely bracket the graphical peak in levels observed around 15 d after delivery. Pooled median vitamin B2 decreased to 124 μg/L at 1–2 mo and reached a nadir of 111 μg/L by 3–4 mo. Over the remainder of the study period, there was a plateau in median milk total vitamin B2 (Figure 2A, B). The pooled median total vitamin B2 intake from 1 to 6 mo was 87 μg/d.

### Comparison with published values

The IOM’s human milk vitamin B2 concentration of 350 μg/L is based on a mean of the concentration measured in milk samples collected from 5 women in 1990 [28] and consideration of data from microbiological analysis of vitamin B2 in 272 samples of human milk published in 1949 [8,29]. Data from other studies using fluorescent measurement techniques were considered invalid because FAD was not detected. The AI for vitamin B2 in infants aged 0–6 mo was calculated assuming a mean human milk consumption of 0.78 L/d, and rounded up from 270 to 300 μg/d. Data from the present study suggest that the median concentration in mature milk is approximately half of the concentration used by the IOM for setting nutrient intake recommendations.

## Vitamin B3

### Background

Vitamin B3 (commonly called niacin) generates the coenzymes NAD and NADP. NAD and NADP are required for oxidative reactions in energy production and play roles in gene expression, cell cycle progression, DNA repair, and neuron development [30]. The vitamin is estimated to be inadequate in 22% of the global population [21]. Maternal dietary intake of the vitamin is correlated with milk concentrations [31].

### Methods

In human milk, vitamin B3 is present as NAD, niacinamide, niacin mononucleotide, nicotinic acid, and niacin riboside (NR). UPLC-MS/MS was used to quantify these vitamers [17].

### Results

**FIGURE 3:**
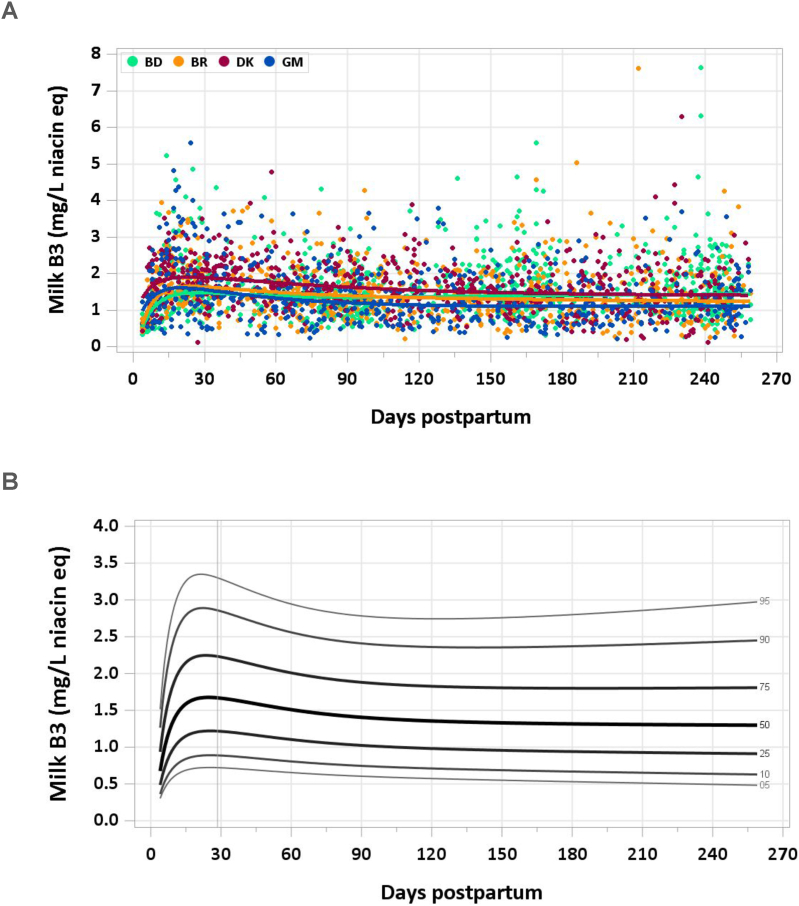
(A) Distribution of human milk vitamin B3 concentrations. (B) Percentile curves for vitamin B3 concentration in human milk.

The total included sample size was 2774 (2252 MILQ, 522 E-MILQ). The median vitamin concentration was 1.43 mg/L at 4–17 d and reached a peak of 1.66 mg/L at 18–31 d. Thereafter, median concentrations fell to 1.58 mg/L at 1–2 mo and 1.38 mg/L at 3–4 mo, plateauing for the remainder of the study period (Figure 3A, B). The pooled median total vitamin B3 intake from 1 to 6 mo was 1.2 mg/d.

### Comparison with published values

The IOM has set the AI for preformed vitamin B3 (excluding the contribution of tryptophan, which can be converted to vitamin B3 in the liver) at 2 mg/d for infants 0–6 mo based on a single study of 23 mothers whose mean milk vitamin B3 was 1.8 mg/L. A microbiological assay was used, and the range of vitamin B3 concentration was 1.2–2.8 mg/L in samples collected between 16 and 244 d of lactation [32]. Although mean milk intake was assumed to be 0.78 L/d, the committee rounded up from 1.4 mg based on the measured milk concentration to 2 mg/d [8]. In the present study, the median vitamin B3 concentration of mature milk was 1.3–1.6 mg/L, below the concentration used by the IOM.

## Pantothenic acid

### Background

Pantothenic acid, as part of coenzyme A and acyl carrier protein, is an essential component of several metabolic reactions, including fatty acid synthesis [33]. Pantothenic acid consists of pantoic acid bound to β-alanine via an amide linkage. The vitamin is found in various plant and animal foods, so deficiency is rare except in severe malnutrition. Consequently, intake and status are rarely measured. The pantothenic acid concentration of milk is correlated with circulating maternal concentrations and dietary intake [34] and is increased by maternal supplementation [5,35].

### Methods

Approximately 85%–90% of pantothenic acid in human milk is in its free form, whereas the remainder is in a bound form [3]. Free pantothenic acid was measured by UPLC-MS/MS.

### Results

**FIGURE 4:**
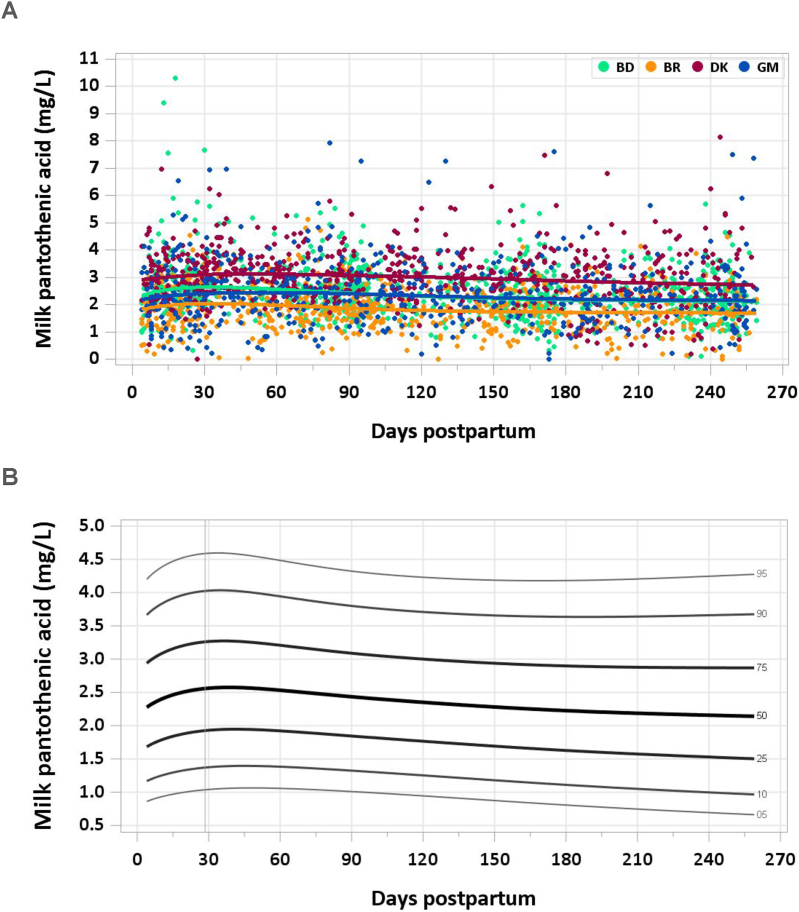
(A) Distribution of human milk pantothenic acid concentrations. (B) Percentile curves for pantothenic acid concentration in human milk.

The total included sample size was 2774 (2252 MILQ, 522 E-MILQ). The median milk concentration of vitamin B9 was 2.31 mg/L at 4–17 d and increased to 2.50 mg/L by 18–31 d. The peak median milk concentration was 2.56 mg/L at 1–2 mo, gradually declining over the remainder of the study period to 2.15 mg/L at 8–8.5 mo (Figure 4A, B). The pooled median total pantothenic acid intake from 1 to 6 mo was 2.0 mg/d.

### Comparison with published values

The IOM has set the AI for pantothenic acid for infants 0–6 mo at 1.7 mg/d based on mean milk pantothenic acid concentrations of 2.2–2.5 mg/L in a summary of 4 studies on 186 women carried out in North America and the United Kingdom [8,35]. Because some women in the studies referenced by the IOM were taking multivitamin supplements containing pantothenic acid, a mean concentration of 2.2 mg/L was multiplied by milk intake of 0.78 L/d to calculate the AI. Data from the MILQ study are consistent with the concentration of 2.2 mg/L pantothenic acid in mature human milk used by the IOM. In addition, 2 studies in China have reported concentrations of 1.8–2.2 mg/L [36,37]. The downward trend in milk pantothenic acid concentration during lactation is consistent with previously published data [37].

## Vitamin B6

### Background

Vitamin B6, as pyridoxal 5’-phosphate (PLP), is a cofactor for >100 enzymes necessary for amino acid metabolism, gluconeogenesis, glycolysis, and hormone regulation [38]. Vitamin B6 encompasses the biologically active, equivalent, and metabolically interconvertible pyridoxal (PL), pyridoxine (PN), and pyridoxamine (PM), as well as their phosphorylated forms. The vitamin is widely distributed in foods, although the content is higher in animal-source foods and some legumes. It has been estimated that 52% of the global population has an inadequate intake of vitamin B6, and the highest prevalence of inadequacy is in Southeast Asia, Latin America, and the Caribbean [21]. The concentration of B6 vitamers in milk is positively correlated with maternal status [39] and responds rapidly to changes in maternal intake [[40], [41], [42]]. Maternal vitamin B6 supplementation increases milk concentrations rapidly [43,44] and in a dose-dependent manner [45].

### Methods

About 75% of vitamin B6 in human milk is in the form of PL, with smaller amounts of PLP, PM, and PN [9,38]. The 4 vitamers of vitamin B6 were quantified using UPLC-MS/MS [46].

### Results

**FIGURE 5:**
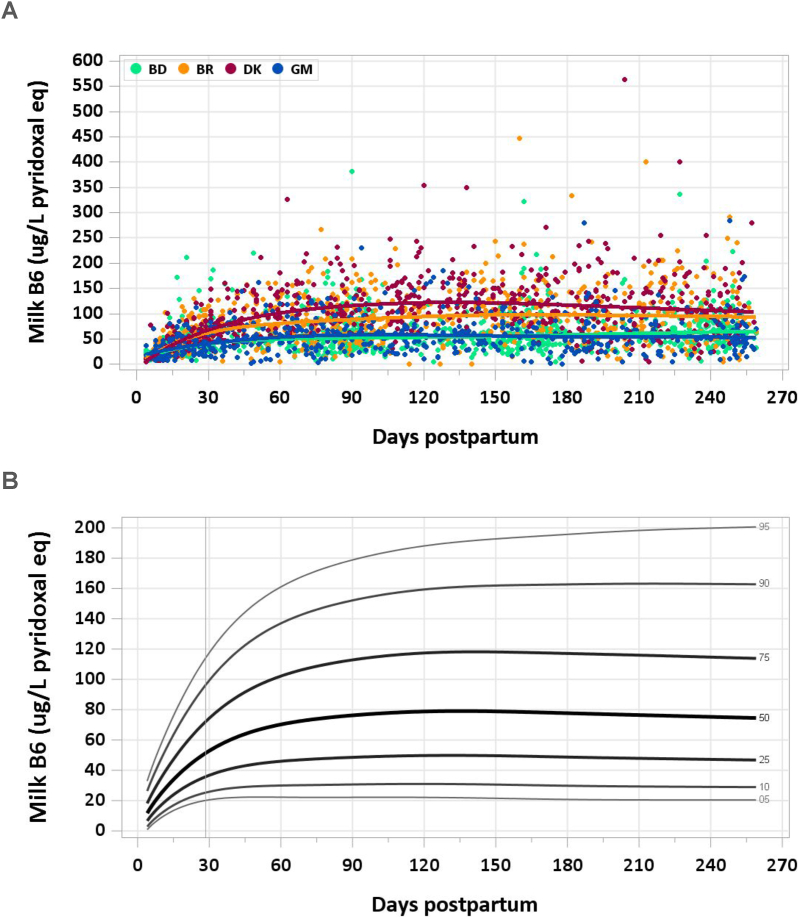
(A) Distribution of human milk vitamin B6 concentrations. (B) Percentile curves for vitamin B6 concentration in human milk.

A total of 3036 samples were analyzed (2462 MILQ, 574 E-MILQ), of which 2 were removed for implausible values (2 MILQ, 0 E-MILQ), and 304 for not meeting inclusion criteria (252 MILQ, 52 E-MILQ), for a total included sample size of 2730 (2208 MILQ, 522 E-MILQ). Median milk vitamin B6 concentration was 23.3 μg/L at 4–17 d and more than doubled to 47.7 μg/L at 18–31 d. The increasing trend continued through a peak of 78.9 μg/L at 4–5 mo, after which there was a gradual decline to 74.7 μg/L by the end of the study period (Figure 5A, B). The pooled median total vitamin B6 intake from 1 to 6 mo was 65 μg/d.

### Comparison with published values

The IOM used a concentration of 130 μg/L vitamin B6 in mature human milk, based on a study of 19 well-nourished women who were supplemented with vitamin B6 or unsupplemented but consuming an intake close to the RDA (2.5 mg/d during lactation) [8]. Samples were collected between 3 wk and 30 mo postpartum, with 5 samples of foremilk taken 5 times per mother over 2 wk. Milk vitamin B6 concentrations were 70–180 μg/L for women consuming <2.5 mg vitamin B6/d, 240 μg/L for intakes 2.5–5 mg/d, and 310 μg/L for intakes >5 mg/d [41]. The IOM set the AI for vitamin B6 in infants 0–6 mo at 100 μg/d, assuming a mean milk intake of 0.78 L/d [8]. Data from the MILQ study suggest that the median vitamin B6 concentration in healthy mothers’ mature milk is ∼60% of the concentration used by the IOM to set infant intake recommendations.

## Biotin

### Background

Biotin acts as a covalently bound coenzyme of carboxylase enzymes that play a crucial role in amino acid metabolism, gluconeogenesis, fatty acid synthesis, and the breakdown of odd-chain fatty acids [38]. In the form of biotinylated histones, biotin is also involved in cell proliferation, regulation of gene expression, and repair of DNA damage [47]. Forms in human milk include biotin as well as its inactive metabolites, bisnorbiotin, and biotin sulfoxide [48]. More than 95% of biotin is present in the skimmed milk fraction [49]. Less than 3% of milk biotin is reversibly bound to macromolecules, and <5% is covalently bound to macromolecules [48]. Biotin is widely distributed in foods. Little is known about the impact of maternal status, intake, or supplementation on milk biotin concentrations.

### Methods

In the MILQ study, free biotin was measured by UPLC-MS/MS for comparability with other data in the literature.

### Results

**FIGURE 6:**
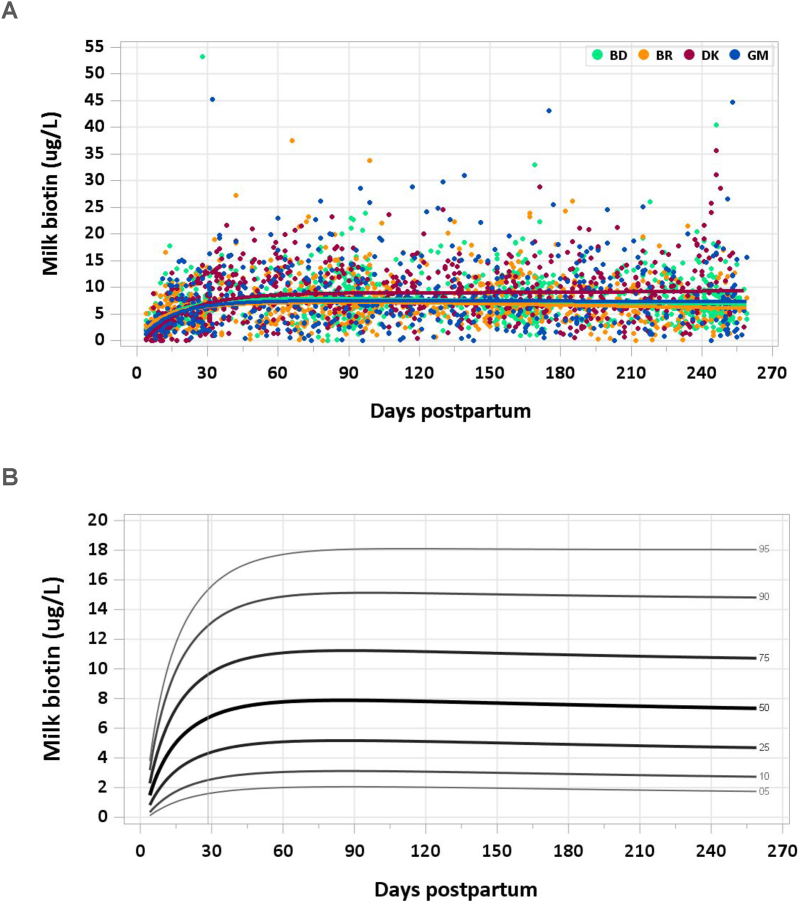
(A) Distribution of human milk biotin concentrations. (B) Percentile curves for biotin concentration in human milk.

A total of 3080 samples were analyzed (2506 MILQ, 574 E-MILQ), of which 4 were removed for implausible values (2 MILQ, 2 E-MILQ), and 304 for not meeting inclusion criteria (253 MILQ, 51 E-MILQ), for a total included sample size of 2772 (2251 MILQ, 521 E-MILQ). Median milk biotin concentration nearly doubled from 4–17 d (3.17 μg/L) to 18–31 d (6.00 μg/L). It peaked at 2–4 mo (7.85–7.86 μg/L), after which it gradually declined to 7.35 μg/L at 8–8.5 mo (Figure 6A, B). The pooled median total biotin intake from 1 to 6 mo was 6.4 μg/d.

### Comparison with published values

The concentration used by the IOM for biotin in human milk is based on the results of 3 studies, 1 in which biotin was measured microbiologically after acid hydrolysis [50] and the other 2 in which biotin was measured by bioassay [51,52]. The range of milk biotin concentration across the studies was 4.5–7.0 μg/L. Using 6 μg/L as the concentration and estimating milk intake to be 0.78 L/d, the IOM set the AI for infants 0–6 mo at 5 μg/d [8]. In 2020, the Nutrition Societies of Germany, Austria, and Switzerland revised recommendations for biotin intake in infants 0–4 mo to 4 μg/d [53] on the basis of human milk biotin concentrations averaging 4.9 μg/L in 2 studies, 1 of which was also used as a reference by the IOM [32,51]. On the basis of the results of the MILQ study, the median biotin concentration in mature milk is 20%–30% higher than the concentration used by the IOM and 50%–60% higher than the German/Austrian/Swiss concentration.

## Vitamin B12

### Background

Vitamin B12 serves as a cofactor in 2 essential enzymatic reactions involved in folate metabolism and DNA synthesis, with deficiency during infancy leading to a range of neurological symptoms and developmental regression [54]. Vitamin B12 is the collective term for cobalt-containing corrinoids, with only the biologically active cobalamins selectively transported into human milk. Because the vitamin is found naturally only in animal-source foods, vegans are at risk of deficiency (unless they consume fortified foods or supplements), and those who avoid or consume small amounts of animal-source foods have lower stores of the vitamin, serum cobalamin, and concentrations in breast milk.

Methylcobalamin is the dominant form of vitamin B12 in human milk, followed by 5΄-deoxyadenosylcobalamin and small amounts of hydroxocobalamin and cyanocobalamin, all strongly bound to haptocorrin [49]. Haptocorrin in human milk is present at 100-fold higher concentrations than in plasma. A relatively strong correlation between concentrations of vitamin B12 in milk and maternal plasma or serum has been shown in previous studies [9,55]. Vitamin B12 concentration in milk is influenced by maternal intake [[56], [57], [58], [59]] as well as supplementation [43,60,61].

Intake of animal-source foods is restricted in many populations due to low income or religious or cultural beliefs, which explains the 39% global prevalence of inadequate vitamin B12 intakes [21]. Poor maternal status during pregnancy reduces liver vitamin B12 stores in the fetus as well as the concentration of the vitamin in breast milk. As a result, their infants are born with low liver stores, and especially if exclusively breastfed, they can develop clinical signs of deficiency around age 3–4 mo [62].

### Methods

Various methods were used in the past to release the vitamin from its binding in plasma with various levels of success, but the present study utilized a competitive chemiluminescence enzyme immunoassay, which avoids the need to remove haptocorrin and has much lower limits of detection [63].

### Results

**FIGURE 7:**
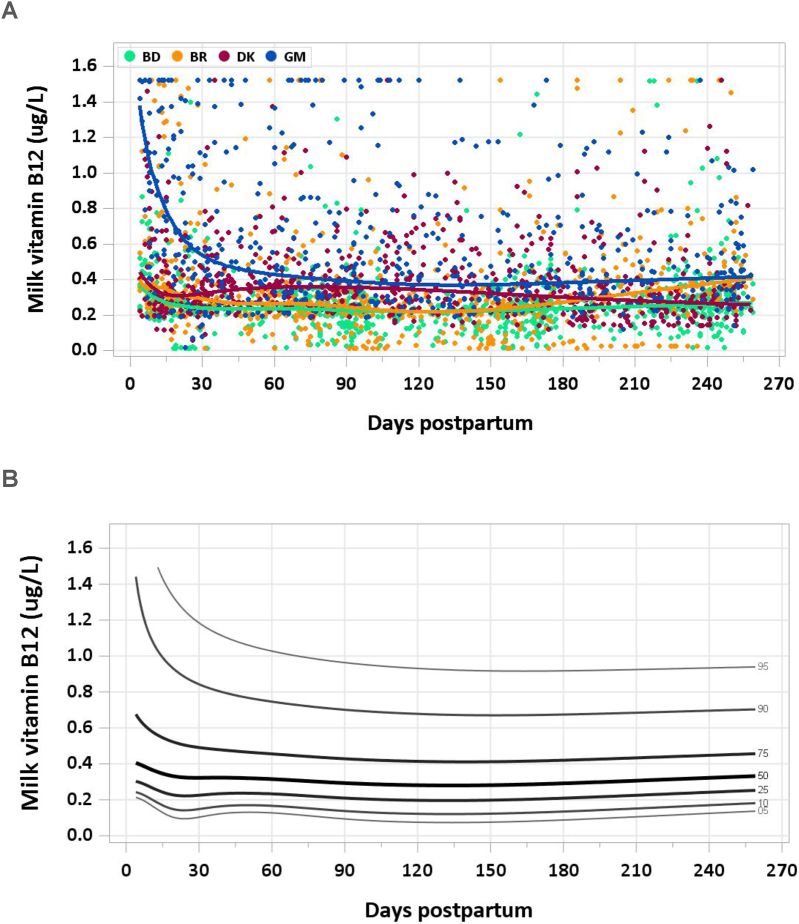
(A) Distribution of human milk vitamin B12 concentrations. (B) Percentile curves for vitamin B12 concentration in human milk.

A total of 3073 samples were analyzed (2502 MILQ, 571 E-MILQ), of which none were removed for implausible values and 306 for not meeting inclusion criteria (255 MILQ, 51 E-MILQ), for a total included sample size of 2767 (2247 MILQ, 520 E-MILQ). However, 1.2% of the measurements were at the assay’s lower limit, and 2.1% at the upper limit, necessitating censored GAMLSS models. Median milk vitamin B12 concentration was 0.416 μg/L (307 pmol/L) at 4–17 d and decreased to 0.305 μg/L (225 pmol/L) at 18–31 d. The median concentration decreased to a nadir of 0.285 μg/L (210 pmol/L) at 1–3.5 mo and increased to 0.314 μg/L (232 pmol/L) by 3.5 to 6 mo (Figure 7A, B). The reason for higher vitamin B12 concentrations in The Gambia, particularly in early lactation, is not known. The pooled median total vitamin B12 intake from 1 to 6 mo was 0.25 μg/d.

### Comparison with published values

The concentration for vitamin B12 in milk used by the IOM is 0.420 μg/L (310 pmol/L), based on a single study in which milk was collected from 4 d to 3 mo lactation from 9 well-nourished, unsupplemented Brazilian mothers (265 samples) [64]. The AI was calculated assuming infant milk intake of 0.78 L/d, but rounding to 0.4 μg/d [8]. Given that vitamin B12 concentration decreases from transitional milk to ∼5 mo of lactation, it is not surprising that the pooled median of 0.302 μg/L (223 pmol/L) from 1 to 8.5 mo in the MILQ study is nearly 30% lower than the IOM estimate of 0.420 μg/L (310 pmol/L) from milk samples collected from early lactation through 3 mo. Furthermore, the radioisotope dilution assay used in the study referenced by the IOM may not have been valid.

A systematic review of vitamin B12 in human milk suggested that the most substantial decrease in concentration occurred during the first months of lactation, reaching a nadir at 3–4 mo [65]. However, the studies included only a few longitudinal measurements. Results of the MILQ study are in accordance with earlier studies, which reported median milk vitamin B12 concentrations in the range of 0.230–0.393 μg/L (170–290 pmol/L) at 3–6 mo [[66], [67], [68]].

## Choline

### Background

The IOM officially recognized choline as an essential nutrient in 1998 [8]. Although it is not a B vitamin, it is often grouped with B vitamins because of similar functions and distribution in food. Choline can be synthesized in the human body, but de novo synthesis alone is insufficient to meet requirements [8]. There has been considerable recent interest in choline requirements and the status of infants due to its essentiality in fetal and infant development [69,70]. Choline may act synergistically with DHA and lutein to improve infants' synapse formation and cognitive performance [71]. There is conflicting evidence on whether maternal dietary intake influences milk choline concentrations [[72], [73], [74], [75]], however, maternal supplementation and status are positively correlated with milk choline [73,74].

### Methods

In the MILQ study, choline was analyzed by UPLC-MS/MS [76]. Over 90% of total choline in milk is water-soluble free choline, phosphocholine, and glycerophosphocholine. The concentrations reported below are the sum of these 3 vitamers. Phosphatidylcholine is a minor contributor.

### Results

**FIGURE 8:**
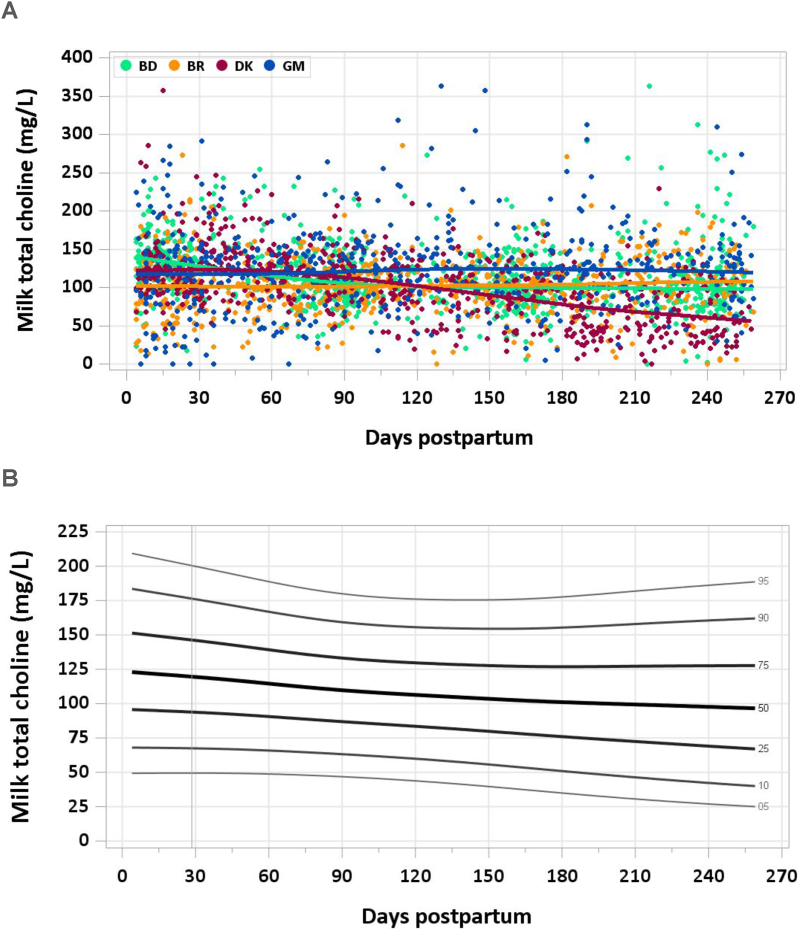
(A) Distribution of human milk choline concentrations. (B) Percentile curves for choline concentration in human.

A total of 3075 samples were analyzed (2500 MILQ, 575 E-MILQ), of which 5 were removed for implausible values (5 MILQ, 0 E-MILQ), and 308 for not meeting inclusion criteria (256 MILQ, 52 E-MILQ), for a total included sample size of 2762 (2239 MILQ, 523 E-MILQ). Median milk total choline concentration was 121 mg/L at 4–17 d and decreased to 112 mg/L at 18–31 d. A downward trend in milk choline concentration continued through the remainder of the study period, with medians of 108 mg/L at 3–4 mo, 102 mg/L at 5–6 mo, and 97 mg/L at 8–8.5 mo (Figure 8A, B). The pooled median total choline intake from 1 to 6 mo was 94 mg/d.

### Comparison with published values

The IOM used a concentration of 160 mg/L based on 2 studies in which choline was measured in human milk [77,78]. Given a mean infant intake volume of 0.78 L/d, the AI was set at 125 mg/d for the first 6 mo. The median concentration of milk choline in the MILQ study was 25%–40% lower than the concentration used by the IOM, depending on the stage of lactation. In a relatively recent comparison of water-soluble forms of choline in milk from Canada and Cambodia analyzed by LC-MS/MS, concentrations were similar in the 2 countries (114 mg/L) and to the MILQ study, supporting the fact that the IOM values are probably too high.

## Discussion

The primary objective of the MILQ study was to develop RVs for nutrient concentrations in the milk of well-nourished mothers during the first 8.5 mo of lactation. Results suggest that milk nutrient concentrations used by the IOM to develop infant intake recommendations may be double to triple the measured concentrations of vitamins B1, B2, and B6 in mature milk, and 1.5 times the measured concentrations of vitamin B6, vitamin B12, and choline. In contrast, milk nutrient concentrations used by the IOM were 80%–90% of those measured in the MILQ study for pantothenic acid and biotin. It is notable that concentrations used by the IOM represent the period from 0 to 6 mo whereas MILQ estimates are from 1 to 6 mo, excluding the earliest period of lactation. Data from the MILQ study demonstrate a peak nutrient concentration before 1 mo lactation for vitamins B2, B3, and B12, which could partially explain the lower milk concentration estimates compared with the IOM values. However, peak nutrient concentrations occurred after 1 mo of lactation for vitamin B1, pantothenic acid, vitamin B6, biotin, and choline.

In the MILQ study, milk volume quantified by deuterium oxide dose-to-mother (or test-weighing in Denmark) together with milk nutrient concentration enabled the calculation of total nutrient intake by the infant at multiple time points over the first 8.5 mo of lactation. The pooled median infant intake of most B vitamins from 1 to 6 mo was less than half of the AI established by the IOM for vitamins B1 and B2, 60%–75% of the AI for vitamins B3, B6, B12, and choline, and 115%–130% of the AI for pantothenic acid and biotin.

Strengths and limitations of the MILQ and E-MILQ studies are discussed in the Introduction and Study Design paper [2], the first in this supplement. The study design was unique in systematically collecting milk from well-nourished women in diverse geographical regions over time points representative of the comprehensive period through 8.5 mo of lactation, and measuring milk volume transferred to the infant in tandem with nutrient concentration.

In conclusion, high precision analytical methods and procedures modified for the milk matrix have enabled the generation of RVs and percentiles for the concentrations of multiple B vitamins in human milk. Most of the MILQ concentrations differ substantially from those reported in the literature due to differences in analytical methods, possible effects of maternal status and diet, and timing and method of sample collection, among other factors. Total daily B-vitamin intakes from the MILQ study ranged from 49% to 128% of existing AIs. The data presented here can be used to evaluate the B-vitamin concentrations in studies collecting human milk samples or to evaluate the impact of maternal interventions. It is hoped that where concentrations differ substantially from values used to set current AIs for infants, those will be improved and used to set EARs.

## Author contributions

The authors’ responsibilities were as follows – LHA, SS-F, SEM, GK, KMF, MMI: designed research; DH, SS-F, FN, ACF: conducted research; DH, JMP: analyzed data; DKD, LHA: wrote the paper; LHA: primary responsibility for the final content; and all authors: read and approved the final manuscript.

## Data availability

Data described in the manuscript, code book, and analytic code will be made available online at a later date.

## Funding

This work was supported by the Bill & Melinda Gates Foundation (OPP1148405/INV-002300, OPP1061055); and USDA intramural funds (2023-51530-025-00D). This paper is published as part of a supplement sponsored by the Bill & Melinda Gates Foundation.

## Conflict of interest

The authors report no conflicts of interest.
