# Supplementary material for: Reference Values for B Vitamins in Human Milk: The Mothers, Infants and Lactation Quality (MILQ) Study
Source: Adv Nutr. 2025 Oct 28;16(Suppl 1):100500. doi: 10.1016/j.advnut.2025.100500 (PMC12673392; doi:10.1016/j.advnut.2025.100500)
Supplement: multimedia component 1 [file mmc1.docx]

**Supplementary Table 1.** Monthly percentile summaries for B vitamin concentration in human milk

| Vitamin B1 | **Age** | **P05** | **P10** | **P25** | **P50** | **P75** | **P90** | **P95** |
| --- | --- | --- | --- | --- | --- | --- | --- | --- |
| (ug/L) |  |  |  |  |  |  |  |  |
|  | 4-17 d | 27.2 | 33.1 | 44.2 | 58.8 | 76.6 | 96.1 | 109.9 |
|  | 18-31 d | 44.6 | 54.3 | 71.7 | 93.1 | 117.8 | 144.4 | 163.3 |
|  | 1-2 m | 53.6 | 66.0 | 86.5 | 110.1 | 136.9 | 166.8 | 189.2 |
|  | 2-3 m | 56.3 | 69.5 | 90.6 | 114.4 | 141.9 | 174.5 | 201.1 |
|  | 3-4 m | 53.9 | 67.0 | 88.1 | 112.1 | 140.5 | 176.3 | 207.1 |
|  | 4-5 m | 49.3 | 62.6 | 84.0 | 108.6 | 138.4 | 177.3 | 212.2 |
|  | 5-6 m | 45.0 | 58.8 | 81.1 | 106.8 | 138.1 | 179.4 | 217.1 |
|  | 6-7 m | 41.9 | 56.5 | 80.2 | 107.2 | 140.0 | 183.0 | 222.0 |
|  | 7-8 m | 39.7 | 55.1 | 80.2 | 108.7 | 142.9 | 187.0 | 226.3 |
|  | 8-8.5 m | 38.4 | 54.4 | 80.4 | 110.0 | 144.9 | 189.5 | 228.8 |
|  |  |  |  |  |  |  |  |  |
|  |  |  |  |  |  |  |  |  |
| Vitamin B2 | **Age** | **P05** | **P10** | **P25** | **P50** | **P75** | **P90** | **P95** |
| (ug/L riboflavin eq) |  |  |  |  |  |  |  |  |
|  | 4-17 d | 92.6 | 105.4 | 129.4 | 161.5 | 202.6 | 252.7 | 292.4 |
|  | 18-31 d | 75.3 | 89.9 | 117.0 | 152.2 | 194.5 | 241.2 | 274.6 |
|  | 1-2 m | 53.4 | 66.6 | 91.6 | 124.0 | 163.0 | 205.8 | 236.2 |
|  | 2-3 m | 45.6 | 57.5 | 80.6 | 112.2 | 151.5 | 195.9 | 227.8 |
|  | 3-4 m | 44.3 | 55.8 | 78.8 | 110.9 | 152.1 | 199.7 | 234.5 |
|  | 4-5 m | 44.1 | 55.5 | 78.7 | 111.8 | 155.0 | 205.6 | 243.1 |
|  | 5-6 m | 44.1 | 55.5 | 79.1 | 113.1 | 158.2 | 211.8 | 251.7 |
|  | 6-7 m | 44.0 | 55.6 | 79.5 | 114.4 | 161.3 | 217.5 | 259.8 |
|  | 7-8 m | 44.0 | 55.6 | 79.9 | 115.6 | 164.0 | 222.7 | 267.2 |
|  | 8-8.5 m | 44.0 | 55.7 | 80.1 | 116.4 | 166.0 | 226.4 | 272.5 |
|  |  |  |  |  |  |  |  |  |
|  |  |  |  |  |  |  |  |  |
| Vitamin B3 | **Age** | **P05** | **P10** | **P25** | **P50** | **P75** | **P90** | **P95** |
| (mg/L niacin eq) |  |  |  |  |  |  |  |  |
|  | 4-17 d | 0.575 | 0.700 | 0.953 | 1.318 | 1.797 | 2.356 | 2.765 |
|  | 18-31 d | 0.720 | 0.885 | 1.214 | 1.667 | 2.235 | 2.872 | 3.324 |
|  | 1-2 m | 0.686 | 0.845 | 1.157 | 1.583 | 2.108 | 2.687 | 3.092 |
|  | 2-3 m | 0.626 | 0.771 | 1.057 | 1.448 | 1.932 | 2.468 | 2.845 |
|  | 3-4 m | 0.587 | 0.725 | 0.998 | 1.375 | 1.847 | 2.377 | 2.755 |
|  | 4-5 m | 0.560 | 0.697 | 0.966 | 1.340 | 1.814 | 2.355 | 2.749 |
|  | 5-6 m | 0.539 | 0.676 | 0.946 | 1.321 | 1.802 | 2.363 | 2.781 |
|  | 6-7 m | 0.520 | 0.659 | 0.932 | 1.310 | 1.800 | 2.385 | 2.833 |
|  | 7-8 m | 0.501 | 0.643 | 0.921 | 1.303 | 1.803 | 2.415 | 2.899 |
|  | 8-8.5 m | 0.487 | 0.632 | 0.913 | 1.299 | 1.807 | 2.441 | 2.954 |
|  |  |  |  |  |  |  |  |  |
|  |  |  |  |  |  |  |  |  |
| Pantothenic acid | **Age** | **P05** | **P10** | **P25** | **P50** | **P75** | **P90** | **P95** |
| (mg/L) |  |  |  |  |  |  |  |  |
|  | 4-17 d | 0.930 | 1.249 | 1.783 | 2.396 | 3.078 | 3.825 | 4.374 |
|  | 18-31 d | 1.020 | 1.352 | 1.906 | 2.536 | 3.237 | 4.001 | 4.563 |
|  | 1-2 m | 1.060 | 1.391 | 1.939 | 2.562 | 3.252 | 4.003 | 4.554 |
|  | 2-3 m | 1.034 | 1.354 | 1.882 | 2.480 | 3.143 | 3.864 | 4.393 |
|  | 3-4 m | 0.975 | 1.285 | 1.801 | 2.387 | 3.038 | 3.746 | 4.267 |
|  | 4-5 m | 0.905 | 1.211 | 1.724 | 2.310 | 2.964 | 3.677 | 4.203 |
|  | 5-6 m | 0.835 | 1.139 | 1.654 | 2.248 | 2.913 | 3.642 | 4.181 |
|  | 6-7 m | 0.770 | 1.074 | 1.595 | 2.201 | 2.885 | 3.637 | 4.193 |
|  | 7-8 m | 0.713 | 1.017 | 1.546 | 2.168 | 2.873 | 3.652 | 4.230 |
|  | 8-8.5 m | 0.674 | 0.978 | 1.513 | 2.147 | 2.869 | 3.669 | 4.264 |
|  |  |  |  |  |  |  |  |  |
|  |  |  |  |  |  |  |  |  |
| Vitamin B6 | **Age** | **P05** | **P10** | **P25** | **P50** | **P75** | **P90** | **P95** |
| (ug/L pyridoxal eq) |  |  |  |  |  |  |  |  |
|  | 4-17 d | 8.8 | 11.7 | 17.4 | 25.4 | 36.0 | 49.0 | 59.1 |
|  | 18-31 d | 18.6 | 23.2 | 32.7 | 46.7 | 65.4 | 87.3 | 103.7 |
|  | 1-2 m | 22.1 | 28.8 | 42.8 | 63.8 | 91.5 | 122.4 | 144.3 |
|  | 2-3 m | 22.1 | 30.3 | 47.5 | 73.9 | 108.6 | 146.0 | 171.6 |
|  | 3-4 m | 22.2 | 30.9 | 49.3 | 77.9 | 115.7 | 156.6 | 184.4 |
|  | 4-5 m | 21.8 | 30.8 | 49.8 | 79.0 | 118.0 | 161.1 | 191.0 |
|  | 5-6 m | 21.1 | 30.1 | 49.1 | 78.2 | 117.6 | 162.4 | 194.5 |
|  | 6-7 m | 20.6 | 29.5 | 48.2 | 77.0 | 116.4 | 163.0 | 197.3 |
|  | 7-8 m | 20.4 | 29.1 | 47.5 | 75.7 | 115.2 | 163.1 | 199.3 |
|  | 8-8.5 m | 20.4 | 28.9 | 46.9 | 74.8 | 114.2 | 162.8 | 200.3 |
|  |  |  |  |  |  |  |  |  |
|  |  |  |  |  |  |  |  |  |
| Biotin | **Age** | **P05** | **P10** | **P25** | **P50** | **P75** | **P90** | **P95** |
| (ug/L) |  |  |  |  |  |  |  |  |
|  | 4-17 d | 0.70 | 1.23 | 2.31 | 3.78 | 5.57 | 7.55 | 8.98 |
|  | 18-31 d | 1.46 | 2.32 | 4.03 | 6.31 | 9.08 | 12.20 | 14.48 |
|  | 1-2 m | 1.88 | 2.89 | 4.87 | 7.48 | 10.67 | 14.32 | 17.03 |
|  | 2-3 m | 2.04 | 3.10 | 5.15 | 7.86 | 11.20 | 15.06 | 17.96 |
|  | 3-4 m | 2.04 | 3.10 | 5.14 | 7.85 | 11.20 | 15.12 | 18.10 |
|  | 4-5 m | 1.99 | 3.03 | 5.06 | 7.75 | 11.10 | 15.06 | 18.09 |
|  | 5-6 m | 1.92 | 2.95 | 4.96 | 7.63 | 10.99 | 14.98 | 18.07 |
|  | 6-7 m | 1.86 | 2.87 | 4.86 | 7.53 | 10.89 | 14.92 | 18.05 |
|  | 7-8 m | 1.80 | 2.80 | 4.77 | 7.43 | 10.80 | 14.86 | 18.05 |
|  | 8-8.5 m | 1.76 | 2.75 | 4.71 | 7.36 | 10.74 | 14.83 | 18.04 |
|  |  |  |  |  |  |  |  |  |
|  |  |  |  |  |  |  |  |  |
| Vitamin B12 | **Age** | **P05** | **P10** | **P25** | **P50** | **P75** | **P90** | **P95** |
| (ug/L) |  |  |  |  |  |  |  |  |
|  | 4-17 d | 0.167 | 0.202 | 0.269 | 0.370 | 0.590 | 1.134 |  |
| Based on | 18-31 d | 0.100 | 0.145 | 0.224 | 0.326 | 0.505 | 0.887 | 1.259 |
| SHASH | 1-2 m | 0.125 | 0.166 | 0.235 | 0.321 | 0.471 | 0.784 | 1.089 |
| Interval | 2-3 m | 0.110 | 0.152 | 0.221 | 0.304 | 0.441 | 0.722 | 0.994 |
| censored | 3-4 m | 0.082 | 0.128 | 0.202 | 0.286 | 0.420 | 0.689 | 0.948 |
| pb(log(x)) | 4-5 m | 0.074 | 0.121 | 0.196 | 0.280 | 0.412 | 0.674 | 0.926 |
| models | 5-6 m | 0.081 | 0.128 | 0.203 | 0.286 | 0.416 | 0.673 | 0.920 |
|  | 6-7 m | 0.097 | 0.144 | 0.218 | 0.300 | 0.428 | 0.682 | 0.925 |
|  | 7-8 m | 0.117 | 0.163 | 0.236 | 0.316 | 0.443 | 0.693 | 0.934 |
|  | 8-8.5 m | 0.132 | 0.177 | 0.249 | 0.329 | 0.454 | 0.702 | 0.941 |
|  |  |  |  |  |  |  |  |  |
|  |  |  |  |  |  |  |  |  |
| Choline | **Age** | **P05** | **P10** | **P25** | **P50** | **P75** | **P90** | **P95** |
| (mg/L) |  |  |  |  |  |  |  |  |
|  | 4-17 d | 49.4 | 67.9 | 95.2 | 122.1 | 150.0 | 181.6 | 207.0 |
|  | 18-31 d | 49.5 | 67.6 | 94.2 | 120.2 | 147.1 | 177.6 | 201.9 |
|  | 1-2 m | 49.2 | 66.7 | 92.2 | 116.9 | 142.4 | 171.1 | 194.0 |
|  | 2-3 m | 47.8 | 64.5 | 88.6 | 111.9 | 135.8 | 162.5 | 183.7 |
|  | 3-4 m | 45.2 | 61.5 | 85.0 | 107.8 | 131.1 | 157.0 | 177.5 |
|  | 4-5 m | 41.5 | 57.6 | 81.5 | 104.7 | 128.5 | 154.8 | 175.6 |
|  | 5-6 m | 36.9 | 52.9 | 77.6 | 102.0 | 127.1 | 154.8 | 176.5 |
|  | 6-7 m | 32.3 | 48.2 | 73.8 | 100.0 | 127.0 | 156.8 | 180.1 |
|  | 7-8 m | 28.3 | 43.8 | 70.4 | 98.3 | 127.4 | 159.6 | 184.6 |
|  | 8-8.5 m | 25.8 | 40.9 | 67.8 | 97.0 | 127.6 | 161.4 | 187.7 |

**Supplementary Table 2**. Median infant B vitamin intake by study visit

|  | **1-3.49 mo** | **3.5-5.99 mo** | **6-8.5 mo** |
| --- | --- | --- | --- |
| **Vitamin B1 (μg/d)** | 90.63 | 87.42 | 74.87 |
| **Vitamin B2 (μg/d)** | 85.33 | 87.26 | 69.75 |
| **Vitamin B3 (mg/d)** | 1.155 | 1.131 | 0.919 |
| **Pantothenic acid (mg/d)** | 1.999 | 1.914 | 1.46 |
| **Vitamin B6 (μg/d)** | 60.55 | 66.26 | 50.99 |
| **Biotin intake (μg/d)** | 6.547 | 6.235 | 4.79 |
| **Vitamin B12 (μg/d)** | 0.27 | 0.24 | 0.21 |
| **Choline (mg/d)** | 93.9 | 88.91 | 72.04 |
